# Utilization and cost of drugs for diabetes and its comorbidities and complications in Kuwait

**DOI:** 10.1371/journal.pone.0268495

**Published:** 2022-06-02

**Authors:** Maryam S. Alowayesh, Syed M. Aljunid, Afaf Al-Adsani, Thamer Alessa, Abdulnabi Alattar, Dherar Alroudhan

**Affiliations:** 1 Department of Pharmacy Practice, School of Pharmacy, Kuwait University, Jabriyah, Kuwait; 2 Department of Health Policy and Management, School of Public Health, Kuwait University, Jabriyah, Kuwait; 3 Al-Sabah Hospital, Ministry of Health, Kuwait City, Kuwait; 4 Jaber Al-Ahmed Hospital, Ministry of Health, Kuwait City, Kuwait; 5 Alamiri Hospital, Ministry of Health, Kuwait City, Kuwait; 6 Mubarak Hospital, Ministry of Health, Kuwait City, Kuwait; Istituto di Ricerche Farmacologiche Mario Negri, ITALY

## Abstract

**Background:**

Diabetes imposes a large burden on countries’ healthcare expenditures. In Kuwait, diabetes prevalence in adults is estimated at 22.0%%—double the worldwide prevalence (9.3%). There is little current data on pharmaceutical costs in Kuwait of managing diabetes and diabetes-related complications and comorbidities.

**Objectives:**

Estimate the utilization and cost of drugs for diabetes and diabetes-related complications and comorbidities in Kuwait for year 2018, as well determinants of costs.

**Methods:**

This cross-sectional study used a multi-stage stratified sampling method. Patients were Kuwaiti citizens with diabetes, aged 18–80, recruited from all six governorates. Physicians collected demographic data, clinical data, and current drug prescription for each patient which was extrapolated for the full year of 2018. A prevalence-based approach and bottom-up costing were used. Data were described according to facility type (primary care vs. hospital). A generalized linear model with log function and normal distribution compared drug costs for patients with and without comorbidities/complications after adjustments for demographic and health confounders (gender, age group, disease duration, and obesity).

**Results:**

Of 1182 diabetes patients, 64.0% had dyslipidemia and 57.7% had hypertension. Additionally, 40.7% had diabetes-related complications, most commonly neuropathy (19.7%). Of all diabetes patients, 85.9% used oral antidiabetics (alone or in combinations), 49.5% used insulin alone or in combinations, and 29.3% used both oral antidiabetics and insulin. The most frequently used oral drug was metformin (75.7%), followed by DPP4 inhibitors (40.2%) and SGLT2 inhibitors (23.8%). The most frequently used injectables were insulin glargine (36.6%), followed by GLP-1 receptor agonists (15.4%). Total annual drug cost for Kuwait’s diabetic population for year 2018 was US$201 million (US$1,236.30 per patient for antidiabetics plus drugs for comorbidities/complications).

**Conclusions:**

Drug costs for treating diabetes and comorbidities/complications accounted for an estimated 22.8% of Kuwait’s 2018 drug expenditures. Comorbidities and complications add 44.7% to the average drug cost per diabetes patient.

## Introduction

Diabetes and its complications are placing an ever-growing strain on the population health and the health expenditures of countries worldwide, including Kuwait. According to the International Diabetes Federation (IDF), over the last two decades, the worldwide prevalence of diabetes has more than tripled, with the 2019 worldwide prevalence of diabetes in adults (aged 20–79) estimated to be 9.3% (463 million people). An additional 1 in 13 (374 million people) have impaired glucose tolerance (pre-diabetes). Half of people with diabetes are unaware they have the disease [1].

In the Middle East and North Africa (MENA) region, which currently comprises 21 countries and territories, including Kuwait, numbers appear worse than the global average. The 2019 IDF Diabetes Atlas estimates that countries in the MENA region have the highest age-adjusted prevalence of diabetes in adults (12.2%, 54.8 million adults aged 20–79) compared to the other six global regions: Africa (4.7%), Europe (6.3%), South & Central America (8.5%), North America and Caribbean (11.1%), Southeast Asia (11.3%), and Western Pacific (11.4%). The IDF ranks Kuwait as the third-highest country for incidence rates (per 100,000 per population per year) of Type 1 diabetes in children aged 0–14 years [1]. Overall diabetes prevalence in adults in Kuwait was 22.0% [1].

A 2015 study estimated that Saudi Arabia had the highest prevalence of diabetes in adults in the MENA region, at 21.8%, followed closely by Jordan and then Kuwait, although percentages were not specified for Jordan and Kuwait [2]. A 2014 study of Kuwaiti citizens estimated that the prevalence of pre-diabetes (usually identified as impaired fasting glucose or impaired glucose tolerance [3]) was 19.4% in Kuwait [4]. Over 40% were unaware of their condition [4].

These high numbers of affected patients represent significant economic impacts on countries’ healthcare systems. In one ten-year span, from 2003–2013, the total global healthcare expenditures for diabetes tripled as the prevalence of diabetes grew and the average expenditure for diabetes increased [5]. In 2019, the IDF estimated the worldwide diabetes-related health expenditure at US$760 billion. The IDF conservatively expects that number to continue to grow 8.6% by 2030 and 11.2% by 2045 [1].

The economic impact of diabetes on MENA countries is expected to rise as the number of people diagnosed with diabetes continues to increase and as the costs of medications climb. In addition, newer antidiabetic medications, such as glucagon-like peptide-1 (GLP-1) receptor agonists and sodium glucose co-transporter 2 (SGLT2) inhibitors, are significantly more expensive than traditional antidiabetic medications, such as metformin, sulfonylureas, and insulin [6].

Patients with diabetes often have severe and long-term complications or comorbidities, which add an additional component to the pharmaceutical costs involved in diabetes treatment [1, 7–13].

Most studies examining the prevalence and costs of treating diabetes focus on regional averages. Few studies have closely examined the pharmaceutical costs of diabetes management, and those that have are outdated and do not account for the recent rise in newer antidiabetic medications, which can be costly [7, 9, 13].

Health services in Kuwait are mainly provided through public healthcare sector through six general hospitals, several specialized hospitals, and primary healthcare centers distributed over the six governorates of Kuwait. Public health services are free for Kuwaiti, GCC nationals, and expatriates working in the Ministry of Health (MoH) and their relatives. Other expatriate residents are required to pay an annual fee of KD 50 ($165) for access to healthcare in public hospitals and clinics and to receive management in the outpatient setting and dispensing most the drugs except for the new expensive branded drugs.

Within Kuwait, it is the responsibility of the Ministry of Finance (MoF) to pay for government funded services. Healthcare in Kuwait is largely financed through oil revenue, which is then distributed to the MoH. The MoH each year provides the MoF with an estimate of the budget required to deliver government-funded healthcare services.

It is then up to the MoF and the Budget Committee within the Parliament to determine if this amount is feasible. Once funds have been pooled, healthcare purchases must use funds to purchase services to meet the healthcare needs of their specific population. There is no social health insurance in Kuwait.

Since health care is offered free of charge only for Kuwaiti nationals by the MoH, this study estimates the utilization and cost of drugs for diabetes and diabetes-related complications and comorbidities for Kuwaiti nationals for year 2018, as well determinants of costs.

## Methods

### Study design

This study was a cross-sectional study using multi-stage stratified sampling. Patients were recruited from all six governorates of Kuwait, including one hospital and two primary care centers from each governorate. Two primary care centers that have diabetes clinics were selected from the list of clinics provided by Ministry of Health (MOH). The centers were chosen if they were in areas that are highly populated by Kuwaiti nationals and if the center’s administration were willing to participate in the study. All eligible patients that came to these clinics on data collection days were approached; therefore, we used convenient sampling, rather than systematic random sampling, because of the shortage of resources.

### Sample size calculation

In the literature, performing sample size calculation for economic analysis is not common. However, since this study looked at factors that affect costs, hence using linear regression models, we performed a sample size calculation according to Maxwell 2000. Using his approach, we needed an N of 1,070. We aimed to recruit 1200, taking into consideration the chance of incomplete forms and missing data.

### Sampling frame

For the sample to be representative of the Kuwaiti population, we used a multi-stage stratified sampling method. The multi-stage refers to the two-stage process; the first stage is the selection of governorates and the second stage is the health facilities i.e the hospital and primary care centers. Stratified means the weight of the strata is accounted for in the sampling, the strata is the governorate. According to the last census in June 2018 from the public authority of civil information, there were 741,648 Kuwaiti adults in all governorates. To determine the weights, we divided the number of Kuwaiti adults in each governorate by the lowest number of Kuwaiti adults found in the smallest governorate (Mubarak Alkabeer). We used the resulting weights to multiply by our target total sample size, to obtain the number for each governorate, as shown in Table 1. Because we didn’t have a priori information on the exact distribution of diabetic patients between primary and secondary care, the number for each governorate was divided equally between the hospital and the two primary care centers, 50% in hospital and 50% in primary.

**Table 1:** Population distribution by Kuwaiti governorate.

Governorate	Kuwaiti Adult population	Sample size
Capital	161,203	260
Hawali	140,894	228
Ahmadi	147,017	238
Farwaniyah	102,610	166
Aljahra	96,568	156
Mubarak Al	93,446	152
Total	741,648	1200

### Patient selection

#### Eligibility criteria

Kuwaiti adults (aged 18 to 80) diagnosed with diabetes were eligible for this study. Both diabetes type 1 and 2 and patients with and without diabetes complications were recruited.

#### Inclusion criteria

Only patients who were diagnosed during or before January 2018 were included so that 1 year of cost data would be available to do the analysis. Only Kuwaiti nationals were included.

#### Exclusion criteria

Non-Kuwaitis were excluded because they do not have the same access to care as Kuwaitis (many branded drugs are for Kuwaitis only) and that would skew the results. Patients who had dementia and were not mentally capable of undergoing the interview were excluded. Females with gestational diabetes were excluded because it is primarily a temporary type of diabetes. Patients above 80 years old were excluded because of the likelihood of having recall issues and because they may have had clinically complex cases due to old age not due to diabetes.

### Data collection

Data were collected using a data collection form created for this study (available in supplements). The data collection form was created through consultation with four consultant diabetologists, a health economist, and five consultants specializing in diabetes complications (a cardiologist, a nephrologist, a neurologist, an ophthalmologist, and a vascular surgeon). Patient clinical data such as diabetes type, onset of diabetes, duration of disease, most recent HbA1c%, comorbidities, and complications were collected by the physician during the outpatient visit by interviewing the patient using the data collection form.

Data regarding each patient’s medication use were collected for the year 2018. We used the prescription prescribed to the patients on their most current visit to their diabetologists. These prescriptions had a 2-month supply, which we then extrapolated for the full year. We extracted each patient’s full medication list from their drug prescription and (if available) dosage strength and dosing frequency.

### Definitions

Comorbidities were defined as other diseases that the patient had that were not caused by diabetes, such as hypertension and hyperlipidemia; complications were considered to be diseases that the patients had that were caused by diabetes, such as nephropathy, retinopathy, neuropathy, peripheral vascular diseases, and cardiovascular and cerebrovascular diseases. The presence of complications and comorbidities was confirmed by the physician through checking the patient’s medical record and by asking the patient questions about their complications, such as whether they visit a specialized doctor to monitor their complications or if they take medications for it, or if they are experiencing some symptoms of these complications.

### Costing

The study used a prevalence-based approach, meaning the average total drug cost per patient from our study sample was multiplied by the estimated total number of patients with diabetes in Kuwait [14]. The estimated prevalence of diabetes in Kuwait was 18.8% in Kuwaiti adults [3], and the Kuwaiti adult population in 2018 (the year of the study) was 741,648. Hence, the number of patients with diabetes in 2018 was estimated at 139,429. We used this number to extrapolate our results to the total diabetic population. The costing in this study was a bottom-up approach, which means every medication use was quantified and added to achieve a total estimate of costs [15]. The cost of drugs is based on MOH drug purchasing price. To calculate the total and per patient cost, the initial prices were converted from Kuwaiti dinar (KD) to US dollars (US$) using an average exchange rate for 2018 of 1 KD = US$3.3106 [16].

### Statistical analysis

We described the data according to facility type (primary care vs. hospital). A generalized linear model with a log function and a normal distribution was performed to explore whether patients with complications and comorbidities have higher drug costs after adjustments for demographic and health confounders (gender, age group, disease duration, and obesity). Patients with no complications and comorbidities (reference category) were compared to those who had both a comorbidity and a complication. Significance level was set at p<0.05. IBM SPSS Statistics version 26 software was used to perform the statistical analysis.

### Ethical considerations

This study is considered Human Subject Research; therefore, an ethical approval has been sought from the Kuwait University (KU) Health Sciences Center (HSC) ethical committee and the Ministry of Health (MOH) ethical committee. This study has been granted ethical approval from the KU-HSC ethical committee on October 8, 2018 (reference number VDR/EC/3411). It was granted approval from the MOH ethical committee on November 4, 2018, (reference number 903/2018). The patients recruited into this study signed a written informed consent.

## Results

### Demographic characteristics

The study included 1182 patients from across Kuwait (Table 2). The mean age was 56.3 (±13.1) years. Most patients were females (65.3%), and most had type 2 diabetes (89.5%). The mean duration of the disease in patients was 13.38 (±8.63) years. The mean HbA1c% was 8.15 (±5.34). Almost two thirds (63.3%) of the patients were obese, with body mass index (BMI) ≥30. Most of the patients (79.0%) were seen by a diabetologist, while 15.1% were seen by a family medicine specialist.

**Table 2:** Patient demographics by facility type.

		Primary care	Hospital	Total	Chi-square / t-test
Parameter	Statistics	N (%)	N (%)	N (%)	p-value
Gender	Male	244 (40.5%)	160 (27.9%)	404 (34.4%)	< 0.001
	Female	359 (59.5%)	413 (72.1%)	772 (65.6%)	
Age (N = 1168)	Mean (SD)	58.72 (10.83)	53.85 (14.68)	56.34 (13.08)	< 0.001
Education	Illiterate	80 (13.6%)	70 (12.8%)	150 (13.2%)	0.396
	Elementary / Intermediate / Secondary	268 (45.6%)	233 (42.4%)	501 (44.1%)	
	Diploma / University	240 (40.8%)	246 (44.8%)	486 (42.7%)	
Marital Status	Single	31 (5.2%)	80 (14.7%)	111 (9.8%)	< 0.001
	Married	482 (81.4%)	357 (65.7%)	839 (73.9%)	
	Divorced / Widowed	79 (13.3%)	106 (19.5%)	185 (16.3%)	
Patient weight (N = 1041)	Mean (SD)	83.2 (15.73)	86.17 (19.31)	84.75 (17.75)	0.007
Patient height (N = 959)	Mean (SD)	161.59 (10.31)	161.6 (10.53)	161.6 (10.42)	0.991
BMI (N = 953)	Mean (SD)	32.08 (5.99)	33.2 (8.56)	32.68 (7.49)	0.021
Obesity	Non-obese (<30)	164 (37%)	186 (36.5%)	350 (36.7%)	< 0.001
	Obese (≥30)	279 (63%)	324 (63.5%)	603 (63.3%)	
Diabetes Type	Type 1	7 (1.2%)	85 (15.4%)	92 (8%)	< 0.001
	Type 2	592 (98.8%)	466 (84.6%)	1058 (92%)	
Duration of disease (N = 1129)	Mean (SD)	12.22 (8.47)	14.49 (8.7)	13.33 (8.65)	< 0.001
Recent HbA1c (N = 1048)	Mean (SD)	8.34 (6.06)	7.94 (4.45)	8.15 (5.34)	0.234
Recent HbA1c (grouped)	< 7.0	164 (30.4%)	132 (26%)	296 (28.2%)	< 0.001
	7.0–9.0	252 (46.7%)	196 (38.6%)	448 (42.7%)	
	>9.0	124 (23%)	180 (35.4%)	304 (29%)	
Smoking status	Never	470 (79.4%)	393 (74.7%)	863 (77.2%)	0.159
	Current	75 (12.7%)	78 (14.8%)	153 (13.7%)	
	Former	47 (7.9%)	55 (10.5%)	102 (9.1%)	
Flu vaccination (Yes / No)	No	450 (75.9%)	402 (71%)	852 (73.5%)	
	Yes	143 (24.1%)	164 (29%)	307 (26.5%)	0.061
Total drugs number (N = 1104)	Mean (SD)	6.8 (2.9)	6.0 (3.4)	6.4 (3.2)	< 0.001
Number of antidiabetic drugs (N = 1089)	Mean (SD)	2.5 (1.2)	2.8 (1.2)	2.7 (1.2)	< 0.001
Physician Job title	Registrar	133 (25.5%)	26 (4.7%)	159 (14.9%)	
	Senior registrar	98 (18.8%)	60 (10.9%)	158 (14.8%)	
	Specialist	213 (40.8%)	56 (10.2%)	269 (25.1%)	
	Senior specialist	20 (3.8%)	225 (41.1%)	245 (22.9%)	
	Consultant	58 (11.1%)	181 (33%)	239 (22.3%)	
Physician Specialty	Diabetologist	383 (65.0%)	513 (94.1%)	896 (79.0%)	< 0.001
	Family Medicine Specialist	139 (23.6%)	32 (5.9%)	171 (15.1%)	
	General Practitioner	67 (11.4%)	0 (0%)	67 (5.9%)	

HbA1c = hemoglobin A1c; Min-Max = minimum-maximum; SD = standard deviation;

### Clinical characteristics

**Fig 1:**
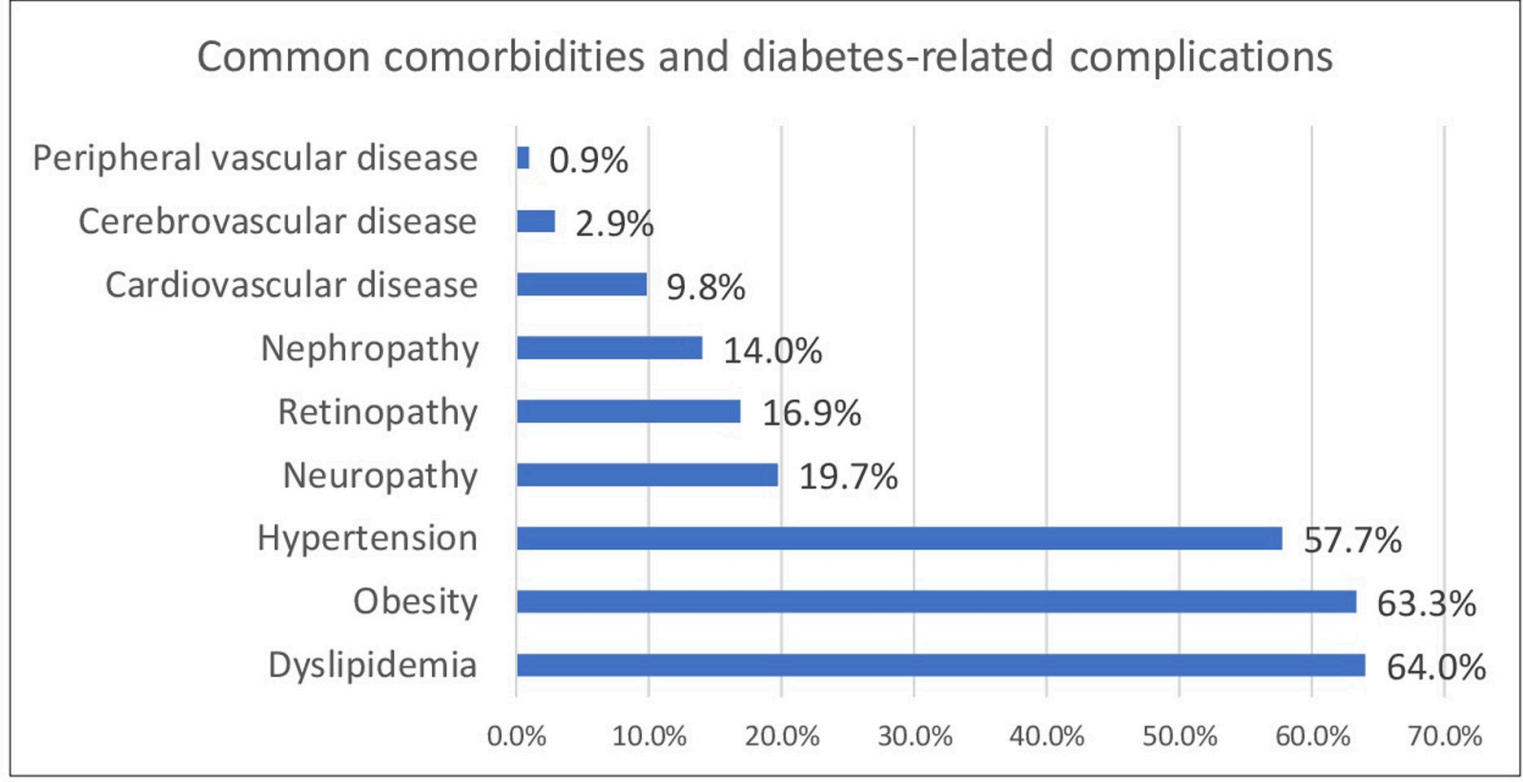
The prevalence of comorbidities and diabetes-related complications in patients with diabetes in Kuwait.

Nearly 4 out of 5 patients with diabetes (79.3%) had a comorbidity, and 40.7% reported having diabetes-related complications. The most common comorbidity was dyslipidemia (64.0%), followed by hypertension (57.7%). Neuropathy was the most prevalent complication (19.7%), followed by retinopathy (16.9%), nephropathy (14.0%), cardiovascular disease (9.8%), cerebrovascular disease (2.9%), and peripheral vascular disease (0.9%) (Fig 1).

### Patients’ treatments and care profile

**Fig 2:**
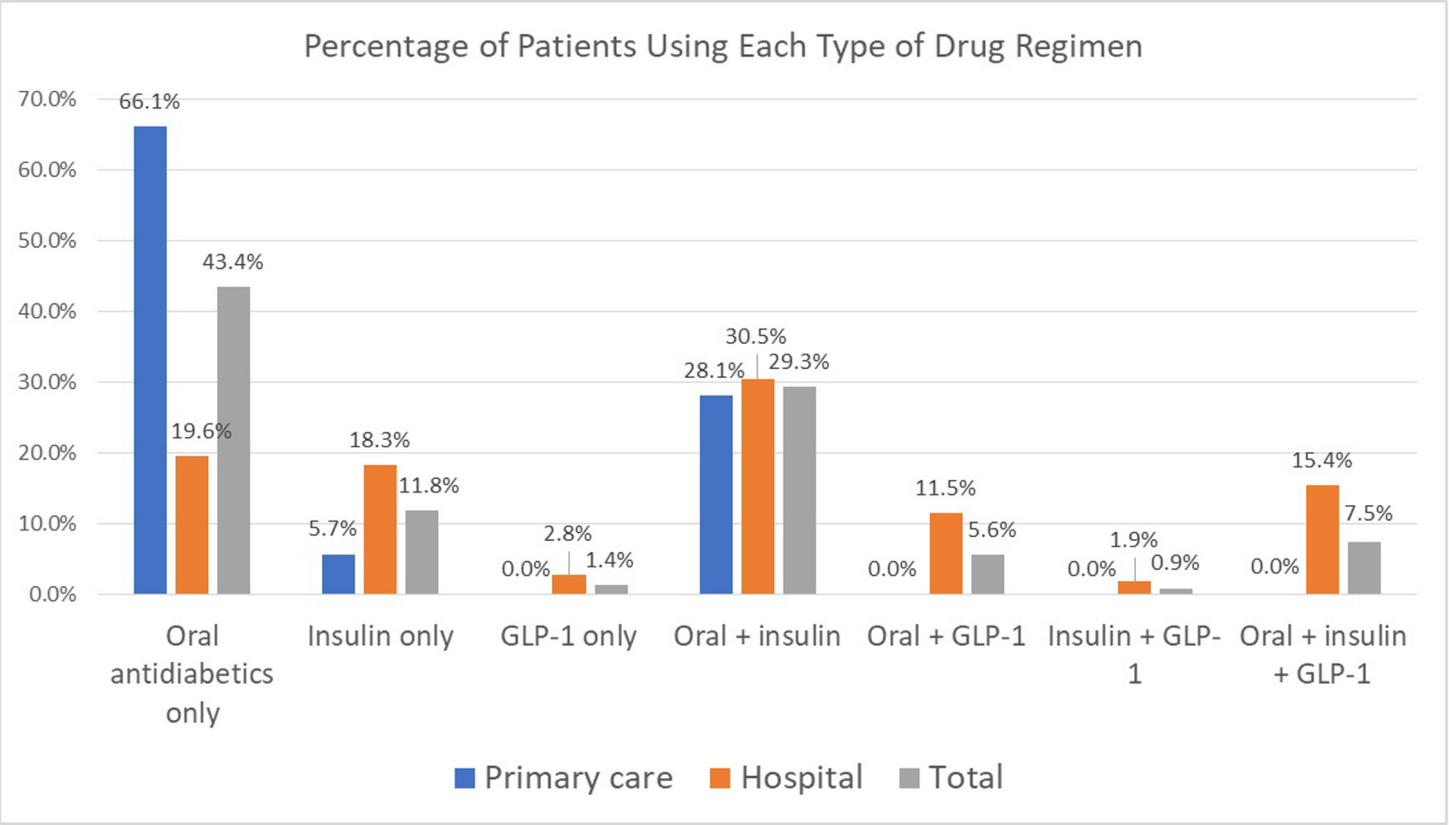
Frequency of different types of drug regimens utilization by facility type in Kuwait.

**Fig 3:**
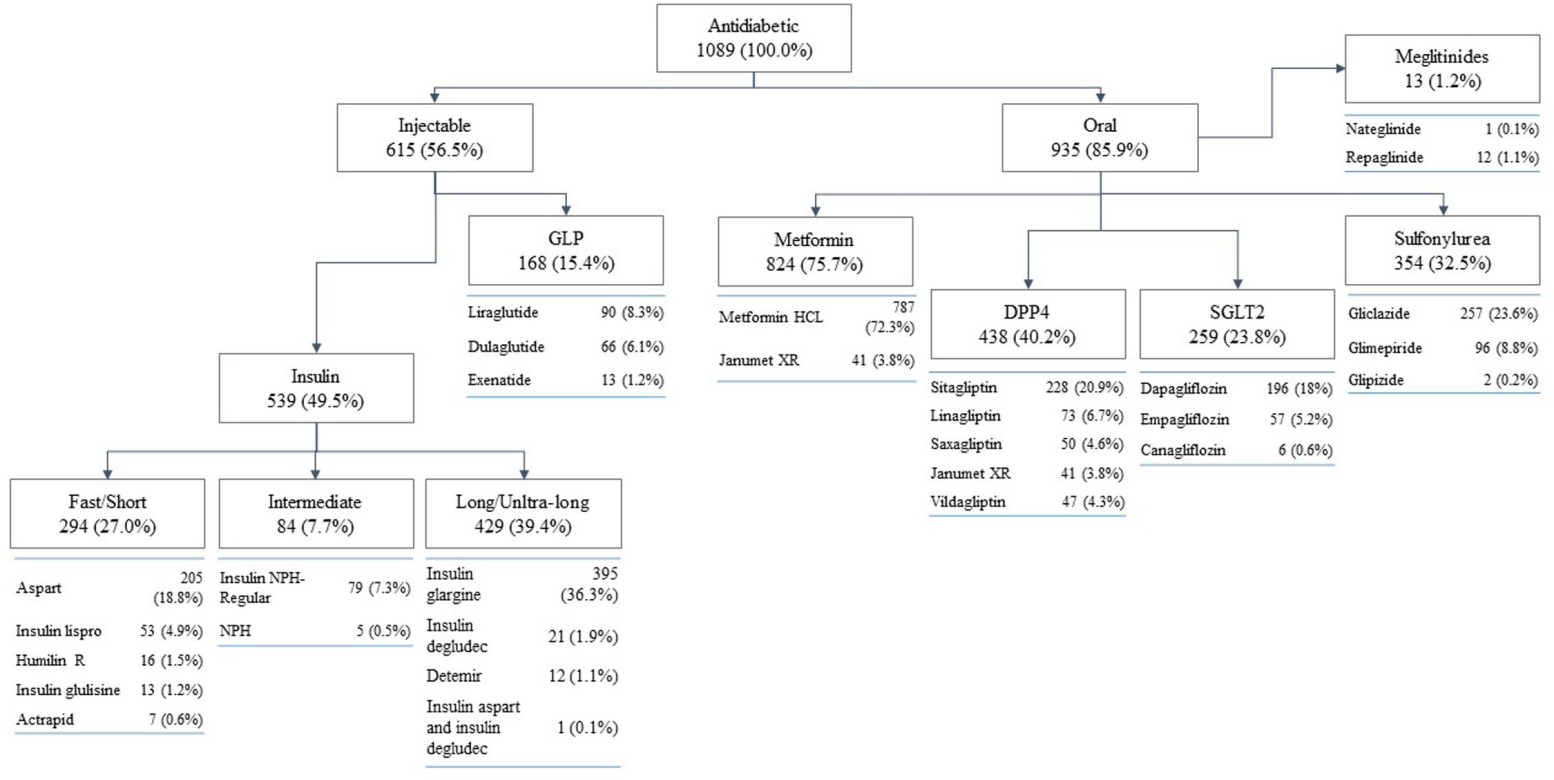
Flowchart showing the utilization of antidiabetics by drug groups.

Of all diabetes patients, 85.9% used oral antidiabetics (both alone and in combination with other therapeutic agents). As for insulin usage, almost half of the patients (49.5%) used it alone or in combinations. Combination of usage of both antidiabetics and insulin was indicated for 29.3% of the patients (Fig 2). The most used oral drug was metformin in all formulations (75.7%), and the most used insulin was insulin glargine (36.3%). The frequency of use of the other antidiabetics were: 23.8% of patients used SGLT2 inhibitors, 15.4% used GLP-1 receptor agonists, and 40.2% used DPP4 inhibitors (S1 Table and Fig 3).

Because of comorbidities and diabetes-related complications, patients were also on chronic medications in addition to their antidiabetic medications; the most common were lipid-lowering drugs and anti-hypertensives (61.3% and 54.1% respectively). Other types of drugs used for complications and comorbidities included anti-platelet medications (29.4%), glucagon (3%), and warfarin (3%).

### Drug costs

The total cost of drugs for year 2018 for the treatment of diabetes and its complications/comorbidities was US$1,357,448 in our study population (1182 patients). The mean average cost of antidiabetics plus drugs for comorbidities/complications was US$1,236.30 per patient per year. Extrapolating these results to the total diabetic population with comorbidities and complications in Kuwait in year 2018, the total drug cost was US$201 million. For patients without any complication or comorbidity, the mean average cost of antidiabetic drugs per year was US$910.50. Extrapolating these results to the total diabetic population with no complications or comorbidites in Kuwait for year 2018, the total drug cost was US$148 million.

### Determinants of cost

Based on regression analysis (Table 3), after controlling for confounding patient characteristics such as gender, age, disease duration, and obesity, the total drug cost (drugs for diabetes and its complications) was 44.7% higher for patients with both comorbidities and complications ([95% CI] = 9.5% - 91.2%, p = 0.009) compared to patients without any comorbidity and complication. The direct cost of only antidiabetic drugs did not change significantly for patients with comorbidities and complications compared to the group of patients without any comorbidity and complication (all p-values > 0.05, refer to Table 3); the increase in drug cost was due to the addition of drugs for the comorbidities/complications.

**Table 3:** Effect of patients’ demographics and clinical characteristics on mean drug cost.

		Antidiabetic Drugs + Drugs for Comorbidities/Complications			Antidiabetic Drugs		
		Ratio	95% CI		Ratio	95% CI	
			Low	High		Low	High
Comorbidity & complication ^a^	Both complications & comorbidities	1.447**	1.095	1.912	1.262	0.987	1.613
Gender ^b^	Female	1.178*	1.013	1.370	1.289**	1.103	1.507
Age group ^c^	≥66 years old	0.643***	0.519	0.796	0.466***	0.368	0.591
	51–65 years old	0.833*	0.711	0.976	0.728***	0.625	0.848
Obesity ^d^	Obese	1.461***	1.237	1.727	1.541***	1.300	1.826
Disease duration (years)		1.016***	1.009	1.024	1.021***	1.013	1.028

Note: * p < .05,

** p < .01,

***p < .001;

^a^ reference category ‘No comorbidity or complication’;

^b^ reference category ‘Male’;

^c^ reference category ‘50- y.o.’;

^d^ reference category ‘Not obese’.

Regression results also showed gender being a significant factor in the average cost per patient both for antidiabetic plus comorbidities/complications drugs and for antidiabetic drugs only. For female patients, the cost of antidiabetic drugs was 28.9% higher than for males ([95% CI] = 10.3% - 50.7%, p = 0.001), and the cost of antidiabetic drugs plus drugs for comorbidities/complications was 17.8% higher than for male patients ([95% CI] = 1.3% - 37.0%, p<0.033).

The average per patient cost also changed for patients belonging to different age groups. The cost of antidiabetic drugs plus drugs for comorbidities/complications was 35.7% lower for patients ≥ 66 years old compared to those ≤ 50 years old ([95% CI] = 20.4% - 48.1%, p<0.001) and was 16.7% lower for patients belonging to age group 51–65 years old compared to the youngest age group ([95% CI] = 2.4% - 28.9%, p = 0.024). Similar results were seen when analyzing the cost of antidiabetic drugs only (without those used for comorbidities/complications); the average combined drug cost was lower for patients ≥ 66 years old, showing a decrease of 53.4% ([95% CI] = 40.9% - 63.2%, p<0.001), and lower for patients aged 51–65 years old, decreasing by 27.2% ([95% CI] = 15.2% - 37.5%, p<0.001).

Patient weight also affected the cost of both antidiabetic drugs plus drugs for comorbidities/complications and for antidiabetics drugs only. Being obese caused a 46.1% ([95% CI] = 23.7% - 72.7%, p<0.001) and 54.1% ([95% CI] = 30.0% - 82.6%, p<0.001) increase in the cost for antidiabetic plus comorbidities/complications drugs and for antidiabetics drugs only, correspondingly compared to non-obese patients.

The average cost of antidiabetic drugs plus comorbidities/complications drugs increased with each year of the disease duration by 1.6% ([95% CI] = 0.90% - 2.4%, p<0.001). For patients only on antidiabetic drugs, the average cost increased by 2.1% with each year of disease duration ([95% CI] = 1.3% - 2.8%, p<0.001).

**Fig 4:**
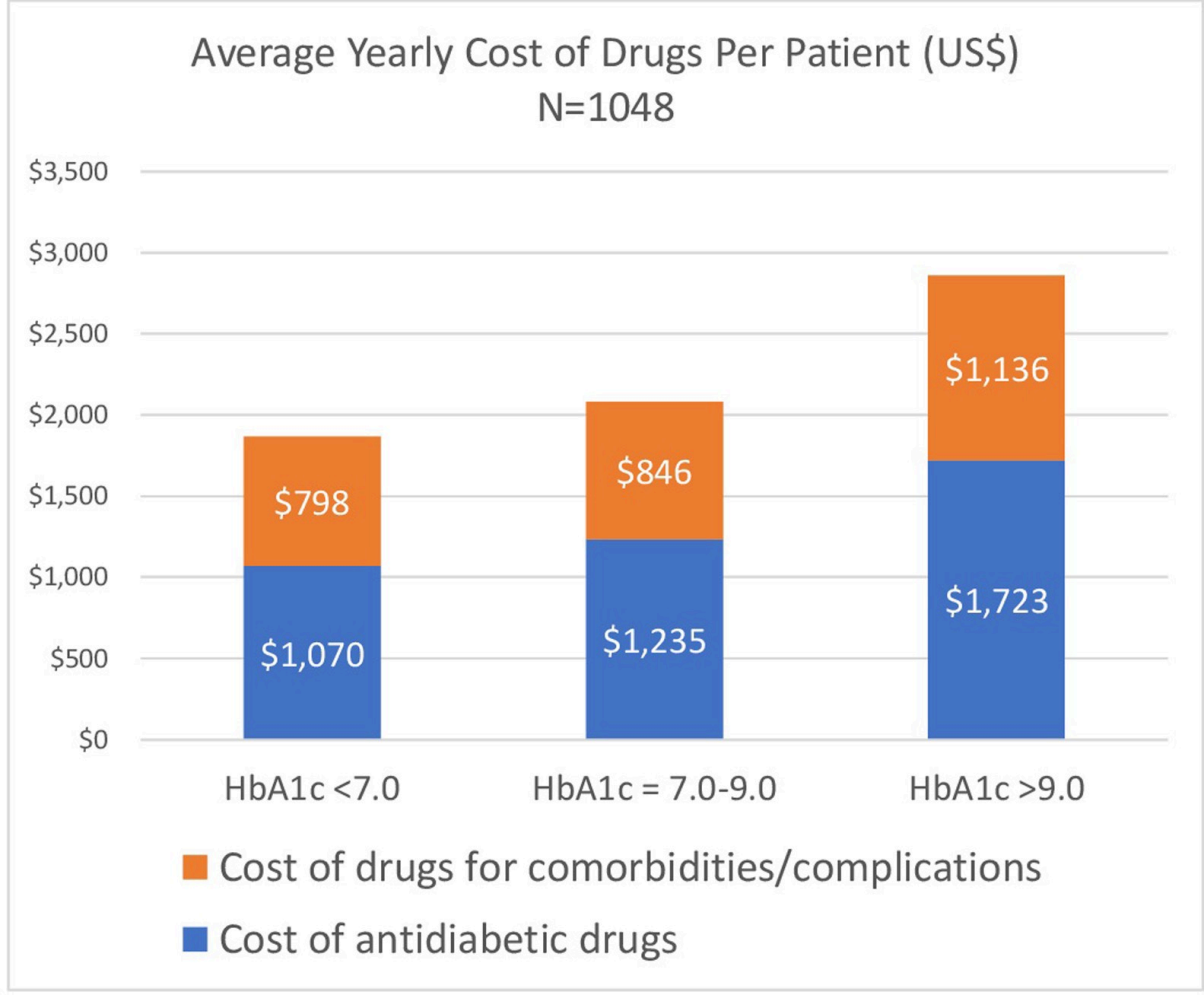
Average price for drugs per year, by patient groups based on HbA1c level (N = 1048).

Patients with HbA1c < 7.0% comprised only 28.2% of the study population. Nearly half (42.7%) of the patients showed HbA1c levels of 7.0–9.0%. Almost a third (29%) of patients had HbA1c levels > 9.0%. Fig 4 shows the average cost of antidiabetics and the average cost of drugs for comorbidities/complications according to glycemic control (HbA1c level).

## Discussion

In 2018, direct pharmaceutical costs for diabetes treatment and diabetes complications and patients’ comorbidities accounted for 22.8lin% of Kuwait’s total drug budget, which is a significant impact. It accounts for 0.1% of the overall GDP of Kuwait. These numbers can be expected to grow with the rising costs of medications and the continuing increase of people in Kuwait diagnosed with diabetes.

Our study showed that 39.2% of patients with diabetes were being treated with medications from the newer antidiabetic drug classes (e.g., GLP-1 receptor agonists or SGLT2 inhibitors), which are significantly more expensive than traditional antidiabetic medications (e.g., metformin, sulfonylureas) [6]. However, even traditional medications continue to rise in cost. Using U.S. costs as an example, the cost of insulin in the U.S. increased sixfold over a 10-year period (2002 to 2012) [17]. Over the 5-year period from 2012 to 2017, the overall cost of diabetes treatment in the U.S. was estimated to have increased by an additional 25%, which was a result of an 11% increase in national prevalence of diagnosed diabetes and a 13% increase in the average cost attributed to diabetes per year in patients with diabetes [18].

When evaluating the pharmaceutical costs of treating diabetes, it is important to understand that the pharmaceutical costs of diabetes management involve more than antidiabetic drugs. Patients with diabetes often have severe and long-term complications or comorbidities, including diabetic ketoacidosis (DKA), hypoglycemia, diabetic eye disease, chronic kidney disease, peripheral neuropathy and diabetic foot complications, and cardiovascular (CV) diseases. Diabetes-associated CV diseases include coronary heart disease, cerebrovascular disease, peripheral artery disease, and congestive heart failure, which can lead to acute coronary syndromes, myocardial infarction, stroke (both ischemic and hemorrhagic), and lower extremity amputations [1]. The percentage of people with diabetes who have diabetes-related complications ranges from country to country. Our study showed that 79.3% of patients with diabetes in Kuwait had at least one comorbidity, and 40.7% reported diabetes-related complications. For comparison, the percentage of patients with diabetes with at least one complication was estimated at 42.8% in Qatar [11], 76.4% in China [12], and 65.1% in Lithuania [19]. The IDF estimates that 32% of people with diabetes also have some type of CV disease, which adds an estimated 50% to the direct costs of diabetes management. CV complications are also associated with higher risk of disability, premature mortality, and loss of productivity, adding to the indirect costs as well [1].

Complications and comorbidities add significantly to the drug cost of diabetes treatment. Our study showed that the total drug cost was 44.7% higher for patients with both comorbidities and complications, 72.0% higher for patients with only complications, and 32.0% higher for patients with only comorbidities. For comparison, in the city of Al-Ain, United Arab Emirates, treatment for patients with diabetes using only diet control and with no complications was 3.2 times higher than the per capita costs for healthcare for patients without diabetes. Diabetes-related microvascular complications increased per-patient costs by 2.2 times, macrovascular complications increased per-patient costs by 6.4 times, and for patients with both micro- and macrovascular complications, costs rose by 9.4 times [7]. In Germany, researchers estimated that the cost of treating patients with diabetes without cardiovascular comorbidities was 1.54 times higher than for treating patients without diabetes. For patients with diabetes with CV comorbidities, the rate of direct costs was 2.77 times higher than for patients without diabetes [8]. In Poland, treatment for diabetes with diabetes-related complications was 5 times greater than the costs of diabetes treatment without complications [9]. And in Brazil, diabetes-related complications increased hospitalizations by 2.4 times while increasing costs by 5.5 times [10].

CV complications lead to the use of additional medications, such as statins, anti-platelet medications, and anti-coagulants. In addition, the American Diabetes Association (ADA) recommends that almost all patients > 40 years of age with diabetes take a statin regardless of CV disease risk level. Our study showed that 78.2% of patients were taking at least one CV-related drug, and 58.7% were taking a statin.

How Kuwait’s diabetes-related healthcare costs compare to the health expenditures for other countries in the MENA region is difficult to compare, due to the relatively small number of studies and differences in study dates, currencies, and health reimbursement systems. One study estimated that in 2010, the health expenditure for diabetes accounted for 14% of the total health expenditure of countries in the Eastern Mediterranean and Middle East, with Saudi Arabia the highest at 21% of total health expenditure [20]. By 2019, the IDF estimated that the countries in the MENA region spent 15.2% of the region’s total health expenditure on diabetes. The only region that spent a higher percentage of their total health expenditure on diabetes was the South & Central America region (19.4%). The European region spent the lowest percentage of health expenditures for diabetes management (8.3%) [1].

Pharmaceutical costs are a significant portion of the healthcare expenditures in countries world-wide. In Iran in 2009, for example, complications accounted for 48.9% of the direct cost of diabetes treatment, and medications accounted for 23.8% of direct costs [13]. In Vietnam, direct medical costs accounted for 51.7% of diabetes treatment costs, with 27.5% of those direct medical costs attributed to medications [21]. In France, pharmacy expenditure represented 21% of all healthcare reimbursements for diabetes [22]. And in the U.S., pharmacy costs represented 12% of all medical costs for antidiabetic agents and diabetes supplies, plus another 18% for medications to treat diabetes-related complications [18].

While drug costs for diabetes and diabetes-related complications are only one aspect of overall healthcare costs for the management of diabetes, quantifying those drug costs and identifying utilization trends is an important step towards understanding the impact of diabetes on the annual healthcare expenditure in Kuwait.

A significant concern in the Kuwaiti population is the number of patients whose diabetes is not controlled, as shown by glycemic levels. In our study, only 28.2% of Kuwaiti patients had HbA1c < 7.0%. Nearly half (42.7%) of the patients had HbA1c levels of 7.0–9.0%. Almost a third (29%) of patients had poor control of their diabetes, with HbA1c levels > 9.0%. By comparison, in the U.S., 50% of patients were estimated to have glycemic control with HbA1c < 7.0, 35.5% of adults had HbA1c of 7.0% - 9.0%, and 14.6% had poor control with HbA1c > 9.0% [23].

## Limitations

A particular strength of this study was the inclusion of data from a high number of patients from all 6 governates of Kuwait and from both hospitals and primary care clinics, which makes the data generalizable to the general Kuwaiti population across all provider settings. A limitation is that, for consistency and access, this study evaluated only patient data for Kuwaiti nationals. Because non-Kuwaitis are estimated to make up 65% of the Kuwait population [24], the average drug costs per non-Kuwaitis may be different due to prescription restrictions. In addition, our study included only patients from the outpatient setting. Further, our data included drug costs, but dosing frequency and dosage strength were not always available for insulins only. Therefore, our evaluations used a calculated average dosage frequency and strength for insulins. Because the data source was written prescriptions, the study did not measure adherence, which could affect real-world results.

## Conclusions

The drug costs of managing diabetes in Kuwait are significant (22.8% of the total drug budget in Kuwait), given the high prevalence of diabetes in Kuwait, the rising costs of drugs, and the increased use of newer, more expensive, classes of antidiabetic drugs. Comorbidities and diabetes-related complications, such as CV disease, add to the cost, with complications and comorbidities responsible for a 44.7% increase in drug cost per patient per year compared to the patients without any comorbidities or complications. Also, based on trials that included data on CV and renal outcomes, the frequently updated international guidelines from the ADA and European Association for the Study of Diabetes (EASD) [25, 26] recommend using a more expensive antidiabetic therapy, namely GLP-1 receptor agonists and SGLT2 inhibitors, in diabetes patients with CV or renal complications or who are at higher risk for development of those diabetes-related complications, for the reduction of such events.

Understanding the true impact of diabetes-related pharmaceutical costs on the health budget of Kuwait is the first step towards prioritizing efforts to manage the diabetes epidemic, reduce costs, and improve outcomes.

## Supporting information

S1 Table Frequency of drug use by facility type in Kuwait (N = 1089). Note: * SITAGLIPTIN+METFORMIN is mentioned twice as it belongs to both Metformin and DPP4 drugs.
(DOCX)

S1 File (PDF)
