# Supplementary material for: Utilization and cost of drugs for diabetes and its comorbidities and complications in Kuwait
Source: PLoS One. 2022 Jun 2;17(6):e0268495. doi: 10.1371/journal.pone.0268495 (PMC9162372; doi:10.1371/journal.pone.0268495)
Supplement: S1 Table — Note: * SITAGLIPTIN+METFORMIN is mentioned twice as it belongs to both Metformin and DPP4 drugs. (DOCX) [file pone.0268495.s001.docx]

|  | **Number of Patients Using:** | **Number of Patients Using:** | **Number of Patients Using:** |
| --- | --- | --- | --- |
|  | **Primary Care**  **N (%)** | **Hospital**  **N (%)** | **Total**  **N (%)** |
| METFORMIN HCL | 473 (84.8%) | 314 (59.1%) | 787 (72.3%) |
| METFORMIN+SITAGLIPTIN | 0 (0%) | 41 (7.7%) | 41 (3.8%) |
| **Overall Metformin** | **473 (84.8%)** | **351 (66.1%)** | **824 (75.7%)** |
| LIRAGLUTIDE | 0 (0%) | 90 (16.9%) | 90 (8.3%) |
| DULAGLUTIDE | 0 (0%) | 66 (12.4%) | 66 (6.1%) |
| EXENATIDE EXTENDED RELEASE | 0 (0%) | 13 (2.4%) | 13 (1.2%) |
| **Overall GLP-1 Receptor Agonists** | **0 (0%)** | **168 (31.6%)** | **168 (15.4%)** |
| DAPAGLIFLOZIN | 104 (18.6%) | 92 (17.3%) | 196 (18%) |
| EMPAGLIFLOZIN | 1 (0.2%) | 56 (10.5%) | 57 (5.2%) |
| CANAGLIFLOZIN | 0 (0%) | 6 (1.1%) | 6 (0.6%) |
| **Overall SGLT2 Inhibitors** | **105 (18.8%)** | **154 (29%)** | **259 (23.8%)** |
| SITAGLIPTIN | 170 (30.5%) | 58 (10.9%) | 228 (20.9%) |
| LINAGLIPTIN | 40 (7.2%) | 33 (6.2%) | 73 (6.7%) |
| SAXAGLIPTIN | 40 (7.2%) | 10 (1.9%) | 50 (4.6%) |
| SITAGLIPTIN+METFORMIN | 0 (0%) | 41 (7.7%) | 41 (3.8%) |
| VILDAGLIPTIN | 44 (7.9%) | 3 (0.6%) | 47 (4.3%) |
| **Overall DPP4 INHIBITORS** | **294 (52.7%)** | **144 (27.1%)** | **438 (40.2%)** |
| NATEGLINIDE | 0 (0%) | 1 (0.2%) | 1 (0.1%) |
| REPAGLINIDE | 1 (0.2%) | 11 (2.1%) | 12 (1.1%) |
| **Overall Meglitinides** | **1 (0.2%)** | **12 (2.3%)** | **13 (1.2%)** |
| GLICLAZIDE | 170 (30.5%) | 87 (16.4%) | 257 (23.6%) |
| GLIMEPIRIDE | 57 (10.2%) | 39 (7.3%) | 96 (8.8%) |
| GLIPIZIDE | 2 (0.4%) | 0 (0%) | 2 (0.2%) |
| **Overall Sulfonylureas** | **229 (41%)** | **125 (23.5%)** | **354 (32.5%)** |
| INSULIN ASPART | 31 (5.6%) | 174 (32.8%) | 205 (18.8%) |
| INSULIN LISPRO | 25 (4.5%) | 28 (5.3%) | 53 (4.9%) |
| HUMILIN R | 15 (2.7%) | 8 (1.5%) | 23 (2.1%) |
| INSULIN GLULISINE | 3 (0.5%) | 10 (1.9%) | 13 (1.2%) |
| **Overall fast/short acting insulin** | **74 (13.3%)** | **220 (41.4%)** | **294 (27%)** |
| INSULIN NPH | 49 (8.8%) | 35 (6.6%) | 84 (7.7%) |
| **Overall intermediate insulin** | **49 (8.8%)** | **35 (6.6%)** | **84 (7.7%)** |
| INSULIN GLARGINE | 122 (21.9%) | 273 (51.4%) | 395 (36.3%) |
| INSULIN DEGLUDEC | 0 (0%) | 21 (4%) | 21 (1.9%) |
| INSULIN DETEMIR | 0 (0%) | 12 (2.3%) | 12 (1.1%) |
| INSULIN ASPART AND INSULIN DEGLUDEC | 1 (0.2%) | 0 (0%) | 1 (0.1%) |
| **Overall long/ultra-long insulin** | **123 (22%)** | **306 (57.6%)** | **429 (39.4%)** |
| **Overall Insulin** | **188 (33.7%)** | **351 (66.1%)** | **539 (49.5%)** |
| **Total (patients who use any antidiabetic or insulin medications)** | **558 (100%)** | **531 (100%)** | **1089 (100%)** |
