# Supplementary material for: Utilization and cost of drugs for diabetes and its comorbidities and complications in Kuwait
Source: PLoS One. 2022 Jun 2;17(6):e0268495. doi: 10.1371/journal.pone.0268495 (PMC9162372; doi:10.1371/journal.pone.0268495)
Supplement: S1 File — (PDF) [file pone.0268495.s002.pdf]

The Economic Burden of Diabetes in Kuwait Study Data Collection Sheet **Patient ID code:**

PHYSICIAN-RELATED DATA (to be filled out by physician)

Job title: ☐ Registrar ☐ Senior registrar ☐ Specialist ☐ Senior specialist ☐ Consultant

Specialty: ☐ Diabetologist ☐ Endocrinologist ☐ Family Medicine Specialist ☐ General Practitioner

DIABETES-RELATED DATA (to be filled out by the physician)

Diabetes type: ☐ Type 1 ☐ Type 2 | Pump pt. : ☐ Yes (*Type: ☐Medtronic® ☐Roche®*) ☐ No

Year of diagnosis: ………………… *OR* Duration of disease (years): ……………………

Hypoglycemia attacks frequency: ……..per/week *OR* ……..per/month *OR* …….per/year

Most recent HbA1c: ………………… | Patient weight: ……………… | Patient height: ………………

COMORBIDITIES ☐None ☐ Hypertension ☐ Dyslipidemia ☐ Others (specify): ……………………………
……………………………..
……………………………

COMPLICATIONS *(ONLY record Interventions that happened in year 2018)*

Retinopathy ☐ None ☐ Yes, if known type and severity (specify): ……………………

-intervention if yes ☐ Photocoagulated (laser) (frequency:……) ☐ Intravitreous injection (frequency:….)
☐ Operated, if known operation (specify): ……………………..

Nephropathy ☐ None ☐ Proteinuria ☐ CKD (stage 3-5) ☐ Dialysis ☐ Transplantation

Neuropathy ☐ None ☐ Peripheral neuropathy ☐ Deformities
☐ Autonomic dysfunction, if known (specify): ………………………….

Cerebrovascular disease ☐ None ☐ stroke, if known (specify): ……………………………

- Level of dependency after stroke ☐ None ☐ Wheel chair ☐ Bed bound

Cardiovascular disease ☐ None ☐ CV event, if known (specify): ……………………..

-intervention if yes ☐ Coronary angiogram study ☐Angioplasty with stent ☐ CABG

Peripheral vascular disease ☐ None ☐ Intermittent claudication ☐ Foot ulceration ☐ Gangrene

-intervention if yes ☐ Angioplasty ☐ Angioplasty with stent ☐ Surgical bypass ☐ Amputation

Please Printout most current Medication Prescription and hand to data collector

Thank you Doctor for your time and effort
